# Supplementary material for: Glycated albumin in the detection of diabetes during COVID-19 hospitalization
Source: PLoS One. 2024 Mar 18;19(3):e0297952. doi: 10.1371/journal.pone.0297952 (PMC10947635; doi:10.1371/journal.pone.0297952)
Supplement: Attachment — Submitted filename: Response to Reviewers PlosONE GA x NDM COVID.docx [file pone.0297952.s003.docx]

Dears Editor and Reviewers,

Thank you for allowing us the chance of re-submitted our manuscript. We believe that we have properly answered the Reviewer’s questions and/or suggestions. The text was amended accordingly. The evaluation and suggestions contributed to improve our manuscript.

The modifications are marked in the main text file.

**Journal requirements:**

**When submitting your revision, we need you to address these additional requirements.**

**1. Please ensure that your manuscript meets PLOS ONE's style requirements, including those for file naming. The PLOS ONE style templates can be found at**

[**https://journals.plos.org/plosone/s/file?id=wjVg/PLOSOne_formatting_sample_main_body.pdf**](https://journals.plos.org/plosone/s/file?id=wjVg/PLOSOne_formatting_sample_main_body.pdf) **and** [**https://journals.plos.org/plosone/s/file?id=ba62/PLOSOne_formatting_sample_title_authors_affiliations.pdf**](https://journals.plos.org/plosone/s/file?id=ba62/PLOSOne_formatting_sample_title_authors_affiliations.pdf)

Thank you for this observation. The manuscript has been amended accordingly.

**2. We note that you have indicated that data from this study are available upon request. PLOS only allows data to be available upon request if there are legal or ethical restrictions on sharing data publicly. For more information on unacceptable data access restrictions, please see** [**http://journals.plos.org/plosone/s/data-availability#loc-unacceptable-data-access-restrictions**](http://journals.plos.org/plosone/s/data-availability#loc-unacceptable-data-access-restrictions)**.**

**In your revised cover letter, please address the following prompts:**

**a) If there are ethical or legal restrictions on sharing a de-identified data set, please explain them in detail (e.g., data contain potentially sensitive information, data are owned by a third-party organization, etc.) and who has imposed them (e.g., an ethics committee). Please also provide contact information for a data access committee, ethics committee, or other institutional body to which data requests may be sent.**

**b) If there are no restrictions, please upload the minimal anonymized data set necessary to replicate your study findings as either Supporting Information files or to a stable, public repository and provide us with the relevant URLs, DOIs, or accession numbers. For a list of acceptable repositories, please see** [**http://journals.plos.org/plosone/s/data-availability#loc-recommended-repositories**](http://journals.plos.org/plosone/s/data-availability#loc-recommended-repositories)**.**

**We will** **update your Data Availability statement on your behalf to reflect the information you provide.**

Thank you, we have found that at our institution there are no ethical or legal restrictions on sharing an anonymized dataset. We added a table in our supplementary material, quoted in the manuscript (Page 23, Line 2) and update our Data Availability statement.

**3. Your ethics statement should only appear in the Methods section of your manuscript. If your ethics statement is written in any section besides the Methods, please delete it from any other section.**

Thank you for this observation. We revised and amended accordingly.

**ANSWERS TO REVIEWERS:**

**5. Review Comments to the Author**

**Please use the space provided to explain your answers to the questions above. You may also include additional comments for the author, including concerns about dual publication, research ethics, or publication ethics. (Please upload your review as an attachment if it exceeds 20,000 characters)**

**Reviewer #1: This manuscript by Fernando Chimela Chume investigated the role of GA in detecting newly diagnosed diabetes during COVID-19 hospitalization in a retrospective study. They enrolled 184 adults from the biobank and get the conclusion that GA higher than 19% is helpful for identify the newly diagnosed diabetes. The major concern from this observation study is lack of the second independent test set for confirm the robust of this index.**

***Answer:*** Thank you for this comment. We followed the Standard for Reporting Diagnostic Accuracy (STARD) initiative guideline to carry out this diagnostic accuracy study. It is recommended to evaluate the index test (i.e., GA) by the most suitable reference standard for the study setting. According to the literature (reference 22 in the manuscript file), HbA1c ≥6.5% is the choice during hospital admission. Therefore, we evaluated the performance of GA to identify patients presenting newly diagnosed diabetes by HbA1c ≥ 6.5% in our study. It is worth to mention that in this present study GA presented performance similar to those seen in outpatient’s studies (reference 27 in the manuscript file). Nevertheless, we agree with Reviewer 1 that comparison with other tests, as we often see in studies with outpatient participants, could provide us with additional information. However, in hospitalized patients tests such as fasting blood glucose and oral glucose tolerance tests are not feasible. Usually, the available test is random blood glucose (RBG) which is susceptible to pre-analytical interferences, including fasting, food intake, and acute illnesses such as COVID-19. We were able to have RBG results for our patients, and we built Receiver Operating Characteristic (ROC) curves to illustrate the performance of RBG and GA as index tests and HbA1c as reference test. The overall diagnostic accuracy for RBG and GA were similar in detecting newly diagnosed diabetes. Nonetheless, RBG is able to detect hyperglycaemia but it is unreliable in diagnosing diabetes in hospitalized patients due to above-mentioned interferences. To avoid misinterpretation, we chose not to show these results in the article.

Newly diagnosed diabetes by HbA1c ≥6.5% (n = 184)

Sensitivity

| 1 - Specificity | RBG: AUC 0.724 (SE: 0.061, 95% CI: 0.605 – 0.843, p <0.001) |
| --- | --- |
| 1 | 1 |
| 0.993464 | 1 |
| 0.986928 | 1 |
| 0.973856 | 1 |
| 0.96732 | 1 |
| 0.96732 | 0.967742 |
| 0.954248 | 0.935484 |
| 0.941176 | 0.903226 |
| 0.921569 | 0.903226 |
| 0.915033 | 0.903226 |
| 0.875817 | 0.903226 |
| 0.856209 | 0.903226 |
| 0.836601 | 0.903226 |
| 0.816993 | 0.903226 |
| 0.803922 | 0.903226 |
| 0.784314 | 0.903226 |
| 0.777778 | 0.903226 |
| 0.745098 | 0.903226 |
| 0.738562 | 0.903226 |
| 0.712418 | 0.870968 |
| 0.705882 | 0.806452 |
| 0.686275 | 0.806452 |
| 0.673203 | 0.806452 |
| 0.653595 | 0.774194 |
| 0.633987 | 0.741935 |
| 0.620915 | 0.741935 |
| 0.607843 | 0.741935 |
| 0.575163 | 0.741935 |
| 0.568627 | 0.741935 |
| 0.542484 | 0.741935 |
| 0.51634 | 0.741935 |
| 0.509804 | 0.709677 |
| 0.496732 | 0.709677 |
| 0.477124 | 0.709677 |
| 0.464052 | 0.709677 |
| 0.431373 | 0.709677 |
| 0.424837 | 0.709677 |
| 0.405229 | 0.709677 |
| 0.392157 | 0.709677 |
| 0.372549 | 0.709677 |
| 0.366013 | 0.709677 |
| 0.339869 | 0.709677 |
| 0.320261 | 0.709677 |
| 0.313725 | 0.709677 |
| 0.300654 | 0.709677 |
| 0.281046 | 0.709677 |
| 0.27451 | 0.709677 |
| 0.267974 | 0.709677 |
| 0.261438 | 0.709677 |
| 0.248366 | 0.677419 |
| 0.24183 | 0.677419 |
| 0.235294 | 0.677419 |
| 0.228758 | 0.612903 |
| 0.222222 | 0.612903 |
| 0.215686 | 0.612903 |
| 0.20915 | 0.612903 |
| 0.202614 | 0.612903 |
| 0.189542 | 0.612903 |
| 0.176471 | 0.612903 |
| 0.169935 | 0.612903 |
| 0.163399 | 0.612903 |
| 0.156863 | 0.612903 |
| 0.156863 | 0.580645 |
| 0.150327 | 0.580645 |
| 0.130719 | 0.548387 |
| 0.124183 | 0.548387 |
| 0.111111 | 0.548387 |
| 0.0980392 | 0.483871 |
| 0.0915033 | 0.451613 |
| 0.0849673 | 0.451613 |
| 0.0784314 | 0.451613 |
| 0.0718954 | 0.419355 |
| 0.0653595 | 0.419355 |
| 0.0653595 | 0.387097 |
| 0.0653595 | 0.354839 |
| 0.0588235 | 0.354839 |
| 0.0588235 | 0.322581 |
| 0.0522876 | 0.290323 |
| 0.0457516 | 0.290323 |
| 0.0392157 | 0.290323 |
| 0.0261438 | 0.290323 |
| 0.0196078 | 0.290323 |
| 0.0196078 | 0.258065 |
| 0.0196078 | 0.225806 |
| 0.0130719 | 0.225806 |
| 0.0130719 | 0.193548 |
| 0.0130719 | 0.16129 |
| 0.0130719 | 0.129032 |
| 0.00653595 | 0.129032 |
| 0 | 0.129032 |
| 0 | 0.0967742 |
| 0 | 0.0645161 |
| 0 | 0.0322581 |
| 0 | 0 |

| 1 - Specificity | GA: AUC 0.739 (SE: 0.049, 95% CI: 0.642 – 0.948, p <0.001) |
| --- | --- |
| 1 | 1 |
| 0.993464 | 1 |
| 0.986928 | 1 |
| 0.980392 | 1 |
| 0.973856 | 1 |
| 0.96732 | 1 |
| 0.960784 | 1 |
| 0.954248 | 1 |
| 0.947712 | 1 |
| 0.941176 | 1 |
| 0.934641 | 1 |
| 0.928105 | 1 |
| 0.921569 | 1 |
| 0.915033 | 1 |
| 0.908497 | 1 |
| 0.901961 | 1 |
| 0.895425 | 1 |
| 0.888889 | 1 |
| 0.882353 | 1 |
| 0.875817 | 1 |
| 0.875817 | 0.967742 |
| 0.869281 | 0.967742 |
| 0.862745 | 0.967742 |
| 0.856209 | 0.967742 |
| 0.849673 | 0.967742 |
| 0.843137 | 0.967742 |
| 0.836601 | 0.967742 |
| 0.830065 | 0.967742 |
| 0.823529 | 0.967742 |
| 0.816993 | 0.967742 |
| 0.810458 | 0.967742 |
| 0.803922 | 0.967742 |
| 0.79085 | 0.967742 |
| 0.784314 | 0.967742 |
| 0.777778 | 0.967742 |
| 0.771242 | 0.967742 |
| 0.764706 | 0.967742 |
| 0.75817 | 0.967742 |
| 0.75817 | 0.935484 |
| 0.751634 | 0.935484 |
| 0.745098 | 0.935484 |
| 0.745098 | 0.903226 |
| 0.738562 | 0.903226 |
| 0.732026 | 0.903226 |
| 0.72549 | 0.903226 |
| 0.718954 | 0.903226 |
| 0.712418 | 0.903226 |
| 0.705882 | 0.903226 |
| 0.69281 | 0.903226 |
| 0.686275 | 0.903226 |
| 0.679739 | 0.903226 |
| 0.679739 | 0.870968 |
| 0.673203 | 0.870968 |
| 0.666667 | 0.870968 |
| 0.660131 | 0.870968 |
| 0.653595 | 0.870968 |
| 0.647059 | 0.870968 |
| 0.647059 | 0.83871 |
| 0.640523 | 0.83871 |
| 0.633987 | 0.83871 |
| 0.633987 | 0.806452 |
| 0.627451 | 0.806452 |
| 0.620915 | 0.806452 |
| 0.614379 | 0.806452 |
| 0.607843 | 0.806452 |
| 0.601307 | 0.806452 |
| 0.594771 | 0.806452 |
| 0.588235 | 0.806452 |
| 0.581699 | 0.806452 |
| 0.575163 | 0.806452 |
| 0.568627 | 0.806452 |
| 0.562092 | 0.806452 |
| 0.555556 | 0.806452 |
| 0.54902 | 0.806452 |
| 0.542484 | 0.806452 |
| 0.535948 | 0.806452 |
| 0.529412 | 0.806452 |
| 0.522876 | 0.806452 |
| 0.51634 | 0.806452 |
| 0.509804 | 0.806452 |
| 0.503268 | 0.806452 |
| 0.496732 | 0.806452 |
| 0.490196 | 0.806452 |
| 0.48366 | 0.806452 |
| 0.470588 | 0.806452 |
| 0.464052 | 0.806452 |
| 0.457516 | 0.806452 |
| 0.457516 | 0.774194 |
| 0.45098 | 0.774194 |
| 0.444444 | 0.774194 |
| 0.437908 | 0.774194 |
| 0.431373 | 0.774194 |
| 0.424837 | 0.774194 |
| 0.418301 | 0.774194 |
| 0.411765 | 0.774194 |
| 0.405229 | 0.774194 |
| 0.398693 | 0.774194 |
| 0.385621 | 0.774194 |
| 0.379085 | 0.774194 |
| 0.372549 | 0.774194 |
| 0.372549 | 0.741935 |
| 0.366013 | 0.741935 |
| 0.359477 | 0.741935 |
| 0.352941 | 0.741935 |
| 0.346405 | 0.741935 |
| 0.339869 | 0.741935 |
| 0.333333 | 0.741935 |
| 0.326797 | 0.741935 |
| 0.320261 | 0.741935 |
| 0.313725 | 0.741935 |
| 0.30719 | 0.741935 |
| 0.300654 | 0.741935 |
| 0.294118 | 0.741935 |
| 0.287582 | 0.741935 |
| 0.281046 | 0.741935 |
| 0.27451 | 0.741935 |
| 0.267974 | 0.741935 |
| 0.261438 | 0.741935 |
| 0.261438 | 0.709677 |
| 0.254902 | 0.709677 |
| 0.248366 | 0.709677 |
| 0.24183 | 0.677419 |
| 0.235294 | 0.677419 |
| 0.235294 | 0.645161 |
| 0.228758 | 0.645161 |
| 0.222222 | 0.645161 |
| 0.222222 | 0.612903 |
| 0.215686 | 0.612903 |
| 0.20915 | 0.612903 |
| 0.202614 | 0.612903 |
| 0.202614 | 0.580645 |
| 0.196078 | 0.580645 |
| 0.196078 | 0.548387 |
| 0.189542 | 0.548387 |
| 0.183007 | 0.548387 |
| 0.176471 | 0.548387 |
| 0.176471 | 0.516129 |
| 0.169935 | 0.516129 |
| 0.163399 | 0.516129 |
| 0.156863 | 0.516129 |
| 0.156863 | 0.483871 |
| 0.150327 | 0.483871 |
| 0.143791 | 0.483871 |
| 0.143791 | 0.451613 |
| 0.143791 | 0.419355 |
| 0.143791 | 0.387097 |
| 0.137255 | 0.387097 |
| 0.130719 | 0.387097 |
| 0.124183 | 0.387097 |
| 0.124183 | 0.354839 |
| 0.124183 | 0.322581 |
| 0.124183 | 0.290323 |
| 0.124183 | 0.258065 |
| 0.117647 | 0.258065 |
| 0.111111 | 0.258065 |
| 0.104575 | 0.258065 |
| 0.0980392 | 0.258065 |
| 0.0915033 | 0.258065 |
| 0.0915033 | 0.225806 |
| 0.0849673 | 0.225806 |
| 0.0784314 | 0.225806 |
| 0.0784314 | 0.193548 |
| 0.0718954 | 0.193548 |
| 0.0653595 | 0.193548 |
| 0.0588235 | 0.193548 |
| 0.0522876 | 0.193548 |
| 0.0522876 | 0.16129 |
| 0.0457516 | 0.16129 |
| 0.0392157 | 0.16129 |
| 0.0326797 | 0.16129 |
| 0.0261438 | 0.16129 |
| 0.0261438 | 0.129032 |
| 0.0261438 | 0.0967742 |
| 0.0196078 | 0.0967742 |
| 0.0130719 | 0.0967742 |
| 0.00653595 | 0.0967742 |
| 0.00653595 | 0.0645161 |
| 0.00653595 | 0.0322581 |
| 0.00653595 | 0 |
| 0 | 0 |

Figure. Receiver operating characteristic (ROC) curve to access the performance of admission RBG and GA to detect newly diagnosed diabetes by HbA1c ≥ 6.5% (n = 184). AUC, area under the curve; CI, confidence interval; HbA1c, glycated haemoglobin; GA, glycated albumin; RBD, random blood glucose; SE, standard error.

**Some other concerns need to be considered:**

**1) GA, HbA1c and the ratio of GA/HbA1c, even with glucose also showed reliable diagnostic value in previous works (PMID: 28393586; PMID: 27386821). But the author only showed the ROC curve of the GA, it should be providing more comprehensive information for authors to compare with these known indexes to get the conclusion that GA is the best one.**

Thank you for this question. We agree that comparison with multiple indexes would give additional information. However, as we mentioned before, we used the most suitable reference standard available for the study setting, in our study was only HbA1c. Laboratorial tests that require fasting are not feasible during acute disease hospitalization.

The use of GA/HbA1c (as an index test) would be interesting, but once HbA1c was the reference test, it would give a mathematically inverted ROC curve due to the use of HbA1c as the denominator in the GA/HbA1c ratio. Below we show the ROC curve for GA/HbA1c ratio, but we chose not show these results in the article. Nevertheless, to present indirectly the mean amplitude of glycaemic excursion in our population we provided GA/HbA1c ratio results in **Table 1** in the article file.

Newly diagnosed diabetes HbA1c ≥6.5%. (n = 184)

Sensitivity

| 1 - Specificity | GA/HbA1c ratio: AUC 0.346 (SE: 0.058, 95% CI: 0.232 – 0.460, p = 0.007) |
| --- | --- |
| 1 | 1 |
| 1 | 0.967742 |
| 1 | 0.935484 |
| 1 | 0.903226 |
| 0.993464 | 0.903226 |
| 0.986928 | 0.903226 |
| 0.986928 | 0.870968 |
| 0.980392 | 0.870968 |
| 0.973856 | 0.870968 |
| 0.96732 | 0.870968 |
| 0.96732 | 0.83871 |
| 0.960784 | 0.83871 |
| 0.954248 | 0.83871 |
| 0.947712 | 0.83871 |
| 0.947712 | 0.806452 |
| 0.941176 | 0.806452 |
| 0.934641 | 0.806452 |
| 0.928105 | 0.806452 |
| 0.928105 | 0.774194 |
| 0.921569 | 0.774194 |
| 0.921569 | 0.741935 |
| 0.921569 | 0.709677 |
| 0.921569 | 0.677419 |
| 0.915033 | 0.677419 |
| 0.908497 | 0.677419 |
| 0.901961 | 0.677419 |
| 0.901961 | 0.645161 |
| 0.901961 | 0.612903 |
| 0.895425 | 0.612903 |
| 0.888889 | 0.612903 |
| 0.882353 | 0.612903 |
| 0.875817 | 0.612903 |
| 0.869281 | 0.612903 |
| 0.862745 | 0.612903 |
| 0.856209 | 0.612903 |
| 0.856209 | 0.580645 |
| 0.849673 | 0.580645 |
| 0.849673 | 0.548387 |
| 0.843137 | 0.548387 |
| 0.836601 | 0.548387 |
| 0.830065 | 0.548387 |
| 0.823529 | 0.548387 |
| 0.816993 | 0.548387 |
| 0.816993 | 0.516129 |
| 0.810458 | 0.516129 |
| 0.810458 | 0.483871 |
| 0.803922 | 0.483871 |
| 0.797386 | 0.483871 |
| 0.79085 | 0.483871 |
| 0.784314 | 0.483871 |
| 0.777778 | 0.483871 |
| 0.771242 | 0.483871 |
| 0.764706 | 0.483871 |
| 0.75817 | 0.483871 |
| 0.751634 | 0.483871 |
| 0.745098 | 0.483871 |
| 0.738562 | 0.483871 |
| 0.72549 | 0.483871 |
| 0.718954 | 0.483871 |
| 0.712418 | 0.483871 |
| 0.705882 | 0.483871 |
| 0.699346 | 0.483871 |
| 0.69281 | 0.483871 |
| 0.686275 | 0.483871 |
| 0.679739 | 0.483871 |
| 0.673203 | 0.483871 |
| 0.673203 | 0.451613 |
| 0.666667 | 0.451613 |
| 0.660131 | 0.451613 |
| 0.653595 | 0.451613 |
| 0.647059 | 0.451613 |
| 0.640523 | 0.451613 |
| 0.633987 | 0.451613 |
| 0.627451 | 0.451613 |
| 0.620915 | 0.451613 |
| 0.614379 | 0.451613 |
| 0.607843 | 0.451613 |
| 0.607843 | 0.419355 |
| 0.601307 | 0.419355 |
| 0.594771 | 0.419355 |
| 0.588235 | 0.419355 |
| 0.581699 | 0.419355 |
| 0.575163 | 0.419355 |
| 0.568627 | 0.419355 |
| 0.562092 | 0.419355 |
| 0.562092 | 0.387097 |
| 0.555556 | 0.387097 |
| 0.54902 | 0.387097 |
| 0.542484 | 0.387097 |
| 0.535948 | 0.387097 |
| 0.529412 | 0.387097 |
| 0.522876 | 0.387097 |
| 0.522876 | 0.354839 |
| 0.51634 | 0.354839 |
| 0.509804 | 0.354839 |
| 0.503268 | 0.354839 |
| 0.496732 | 0.354839 |
| 0.490196 | 0.354839 |
| 0.48366 | 0.354839 |
| 0.477124 | 0.354839 |
| 0.470588 | 0.354839 |
| 0.470588 | 0.322581 |
| 0.464052 | 0.322581 |
| 0.464052 | 0.290323 |
| 0.457516 | 0.290323 |
| 0.45098 | 0.290323 |
| 0.45098 | 0.258065 |
| 0.444444 | 0.258065 |
| 0.437908 | 0.258065 |
| 0.431373 | 0.258065 |
| 0.424837 | 0.258065 |
| 0.418301 | 0.258065 |
| 0.411765 | 0.258065 |
| 0.405229 | 0.258065 |
| 0.398693 | 0.258065 |
| 0.392157 | 0.258065 |
| 0.385621 | 0.258065 |
| 0.379085 | 0.258065 |
| 0.372549 | 0.258065 |
| 0.366013 | 0.258065 |
| 0.359477 | 0.258065 |
| 0.352941 | 0.258065 |
| 0.346405 | 0.258065 |
| 0.339869 | 0.258065 |
| 0.333333 | 0.258065 |
| 0.333333 | 0.225806 |
| 0.333333 | 0.193548 |
| 0.326797 | 0.193548 |
| 0.320261 | 0.193548 |
| 0.313725 | 0.193548 |
| 0.30719 | 0.193548 |
| 0.300654 | 0.193548 |
| 0.300654 | 0.16129 |
| 0.300654 | 0.129032 |
| 0.294118 | 0.129032 |
| 0.287582 | 0.129032 |
| 0.281046 | 0.129032 |
| 0.27451 | 0.129032 |
| 0.267974 | 0.129032 |
| 0.261438 | 0.129032 |
| 0.254902 | 0.129032 |
| 0.248366 | 0.129032 |
| 0.24183 | 0.129032 |
| 0.235294 | 0.129032 |
| 0.228758 | 0.129032 |
| 0.222222 | 0.129032 |
| 0.215686 | 0.129032 |
| 0.20915 | 0.129032 |
| 0.202614 | 0.129032 |
| 0.196078 | 0.129032 |
| 0.196078 | 0.0967742 |
| 0.189542 | 0.0967742 |
| 0.189542 | 0.0645161 |
| 0.183007 | 0.0645161 |
| 0.176471 | 0.0645161 |
| 0.169935 | 0.0645161 |
| 0.163399 | 0.0645161 |
| 0.156863 | 0.0645161 |
| 0.150327 | 0.0645161 |
| 0.143791 | 0.0645161 |
| 0.137255 | 0.0645161 |
| 0.130719 | 0.0645161 |
| 0.124183 | 0.0645161 |
| 0.117647 | 0.0645161 |
| 0.111111 | 0.0645161 |
| 0.104575 | 0.0645161 |
| 0.0980392 | 0.0645161 |
| 0.0915033 | 0.0645161 |
| 0.0849673 | 0.0645161 |
| 0.0849673 | 0.0322581 |
| 0.0784314 | 0.0322581 |
| 0.0718954 | 0.0322581 |
| 0.0653595 | 0.0322581 |
| 0.0588235 | 0.0322581 |
| 0.0522876 | 0.0322581 |
| 0.0522876 | 0 |
| 0.0457516 | 0 |
| 0.0392157 | 0 |
| 0.0326797 | 0 |
| 0.0261438 | 0 |
| 0.0196078 | 0 |
| 0.0130719 | 0 |
| 0.00653595 | 0 |
| 0 | 0 |

| 1 - Specificity | GA: AUC 0.739 (SE: 0.049, 95% CI: 0.642 – 0.948, p <0.001) |
| --- | --- |
| 1 | 1 |
| 0.993464 | 1 |
| 0.986928 | 1 |
| 0.980392 | 1 |
| 0.973856 | 1 |
| 0.96732 | 1 |
| 0.960784 | 1 |
| 0.954248 | 1 |
| 0.947712 | 1 |
| 0.941176 | 1 |
| 0.934641 | 1 |
| 0.928105 | 1 |
| 0.921569 | 1 |
| 0.915033 | 1 |
| 0.908497 | 1 |
| 0.901961 | 1 |
| 0.895425 | 1 |
| 0.888889 | 1 |
| 0.882353 | 1 |
| 0.875817 | 1 |
| 0.875817 | 0.967742 |
| 0.869281 | 0.967742 |
| 0.862745 | 0.967742 |
| 0.856209 | 0.967742 |
| 0.849673 | 0.967742 |
| 0.843137 | 0.967742 |
| 0.836601 | 0.967742 |
| 0.830065 | 0.967742 |
| 0.823529 | 0.967742 |
| 0.816993 | 0.967742 |
| 0.810458 | 0.967742 |
| 0.803922 | 0.967742 |
| 0.79085 | 0.967742 |
| 0.784314 | 0.967742 |
| 0.777778 | 0.967742 |
| 0.771242 | 0.967742 |
| 0.764706 | 0.967742 |
| 0.75817 | 0.967742 |
| 0.75817 | 0.935484 |
| 0.751634 | 0.935484 |
| 0.745098 | 0.935484 |
| 0.745098 | 0.903226 |
| 0.738562 | 0.903226 |
| 0.732026 | 0.903226 |
| 0.72549 | 0.903226 |
| 0.718954 | 0.903226 |
| 0.712418 | 0.903226 |
| 0.705882 | 0.903226 |
| 0.69281 | 0.903226 |
| 0.686275 | 0.903226 |
| 0.679739 | 0.903226 |
| 0.679739 | 0.870968 |
| 0.673203 | 0.870968 |
| 0.666667 | 0.870968 |
| 0.660131 | 0.870968 |
| 0.653595 | 0.870968 |
| 0.647059 | 0.870968 |
| 0.647059 | 0.83871 |
| 0.640523 | 0.83871 |
| 0.633987 | 0.83871 |
| 0.633987 | 0.806452 |
| 0.627451 | 0.806452 |
| 0.620915 | 0.806452 |
| 0.614379 | 0.806452 |
| 0.607843 | 0.806452 |
| 0.601307 | 0.806452 |
| 0.594771 | 0.806452 |
| 0.588235 | 0.806452 |
| 0.581699 | 0.806452 |
| 0.575163 | 0.806452 |
| 0.568627 | 0.806452 |
| 0.562092 | 0.806452 |
| 0.555556 | 0.806452 |
| 0.54902 | 0.806452 |
| 0.542484 | 0.806452 |
| 0.535948 | 0.806452 |
| 0.529412 | 0.806452 |
| 0.522876 | 0.806452 |
| 0.51634 | 0.806452 |
| 0.509804 | 0.806452 |
| 0.503268 | 0.806452 |
| 0.496732 | 0.806452 |
| 0.490196 | 0.806452 |
| 0.48366 | 0.806452 |
| 0.470588 | 0.806452 |
| 0.464052 | 0.806452 |
| 0.457516 | 0.806452 |
| 0.457516 | 0.774194 |
| 0.45098 | 0.774194 |
| 0.444444 | 0.774194 |
| 0.437908 | 0.774194 |
| 0.431373 | 0.774194 |
| 0.424837 | 0.774194 |
| 0.418301 | 0.774194 |
| 0.411765 | 0.774194 |
| 0.405229 | 0.774194 |
| 0.398693 | 0.774194 |
| 0.385621 | 0.774194 |
| 0.379085 | 0.774194 |
| 0.372549 | 0.774194 |
| 0.372549 | 0.741935 |
| 0.366013 | 0.741935 |
| 0.359477 | 0.741935 |
| 0.352941 | 0.741935 |
| 0.346405 | 0.741935 |
| 0.339869 | 0.741935 |
| 0.333333 | 0.741935 |
| 0.326797 | 0.741935 |
| 0.320261 | 0.741935 |
| 0.313725 | 0.741935 |
| 0.30719 | 0.741935 |
| 0.300654 | 0.741935 |
| 0.294118 | 0.741935 |
| 0.287582 | 0.741935 |
| 0.281046 | 0.741935 |
| 0.27451 | 0.741935 |
| 0.267974 | 0.741935 |
| 0.261438 | 0.741935 |
| 0.261438 | 0.709677 |
| 0.254902 | 0.709677 |
| 0.248366 | 0.709677 |
| 0.24183 | 0.677419 |
| 0.235294 | 0.677419 |
| 0.235294 | 0.645161 |
| 0.228758 | 0.645161 |
| 0.222222 | 0.645161 |
| 0.222222 | 0.612903 |
| 0.215686 | 0.612903 |
| 0.20915 | 0.612903 |
| 0.202614 | 0.612903 |
| 0.202614 | 0.580645 |
| 0.196078 | 0.580645 |
| 0.196078 | 0.548387 |
| 0.189542 | 0.548387 |
| 0.183007 | 0.548387 |
| 0.176471 | 0.548387 |
| 0.176471 | 0.516129 |
| 0.169935 | 0.516129 |
| 0.163399 | 0.516129 |
| 0.156863 | 0.516129 |
| 0.156863 | 0.483871 |
| 0.150327 | 0.483871 |
| 0.143791 | 0.483871 |
| 0.143791 | 0.451613 |
| 0.143791 | 0.419355 |
| 0.143791 | 0.387097 |
| 0.137255 | 0.387097 |
| 0.130719 | 0.387097 |
| 0.124183 | 0.387097 |
| 0.124183 | 0.354839 |
| 0.124183 | 0.322581 |
| 0.124183 | 0.290323 |
| 0.124183 | 0.258065 |
| 0.117647 | 0.258065 |
| 0.111111 | 0.258065 |
| 0.104575 | 0.258065 |
| 0.0980392 | 0.258065 |
| 0.0915033 | 0.258065 |
| 0.0915033 | 0.225806 |
| 0.0849673 | 0.225806 |
| 0.0784314 | 0.225806 |
| 0.0784314 | 0.193548 |
| 0.0718954 | 0.193548 |
| 0.0653595 | 0.193548 |
| 0.0588235 | 0.193548 |
| 0.0522876 | 0.193548 |
| 0.0522876 | 0.16129 |
| 0.0457516 | 0.16129 |
| 0.0392157 | 0.16129 |
| 0.0326797 | 0.16129 |
| 0.0261438 | 0.16129 |
| 0.0261438 | 0.129032 |
| 0.0261438 | 0.0967742 |
| 0.0196078 | 0.0967742 |
| 0.0130719 | 0.0967742 |
| 0.00653595 | 0.0967742 |
| 0.00653595 | 0.0645161 |
| 0.00653595 | 0.0322581 |
| 0.00653595 | 0 |
| 0 | 0 |

Figure. Receiver operating characteristic (ROC) curve to access the performance of admission GA/HbA1c ratio and GA to detect newly diagnosed diabetes by HbA1c ≥6.5% (n = 184). AUC, area under the curve; CI, confidence interval; HbA1c, glycated haemoglobin; GA, glycated albumin; RBD, random blood glucose; SE, standard error.

**2) Even this works carried out in the COVID-19 patients, I think the promising index for early diagnosis of newly diabetes is also important in those subjects without COVID-19 infection. The following question is how does the SARS-COV2 virus influent the level of GA? And What's the differential diagnosis value of GA for newly diabetes with other infectious disease?**

Thank you for these questions. During the COVID-19 pandemic, several studies have reported that patients with diabetes mellitus (DM) have a higher risk of severe SARS-CoV-2 infection. Many reasons may explain this susceptibility, including the direct effect of hyperglycaemia in the immune system. However, this disease is characterized by a variety of clinical manifestations ranging from asymptomatic to severe symptoms that may contribute to glucose imbalance. In addition, the influence of genetic variations on clinical outcomes must be considered (References 1 to 4 in the manuscript file). The mechanism by how SARS-CoV-2 affects glucose and GA levels is unclear. The evaluation of the influence of SARS-CoV-2 on the blood GA level is out of the scope of the present study, but is clinically relevant, and it is our understanding that specific studies designed for this purpose are necessary.

As far as we know, this is the first report to evaluate the GA performance in detecting newly diagnosed diabetes in acute ill (particularly COVID-19) individuals during hospital admission. Though data about GA performance in the diagnosis of diabetes in outpatients have been accumulating worldwide, there are no data on hospital admission subjects. We believe the test might be useful in other acute infectious diseases, but further studies are necessary for accurate conclusions about this issue. We amended the text to clarify these points (**Page 16, paragraph 1, lines 13 – 19**).

**3) The prospective study was carried out in COVID-19 patients from March 2020 to December 2020. It should be Original virus strain, have you compared to the newly virus strain with the original virus to explore the diagnostic value of GA of newly diabetes with COVID-19 infection?**

Thank you for this comment. We carried out our study retrospectively in samples available from a biobank in the first year of pandemic, before COVID-19 vaccines to reduce confounding factors, especially if SARS-CoV-2 influences on blood glucose and GA levels. However, we understand that additional studies in new scenarios with new virus strains are needed.

**4) It was reported the BMI correlates positively with HbA1c and negatively with GA. So the HbA1c may be more effective in obese and GA in nonobese individuals (PMID: 35783481; PMID: 17434227). In your study the average BMI of the subject is 29.4 (25.7, 34.5) that across the overweight and obesity group. How to consider the BMI in affecting the diagnostic value of GA?**

Thank you for this comment. We performed the correlations between HbA1c, GA, and BMI. The correlations were not significant (r = 0.020 and 0.029; p >0.100; results not shown in the article). These results are similar to those reported in our previous study (Ref 28 in the manuscript file).

**Reviewer #2: 1- As they stated in their limitations section only once measurement for HbA1c and GA is the main limitation of the current study. Unfortunately, this has a big impact on the data validity.**

Thank you for this comment. This is a limitation of all cross-sectional analysis where longitudinal analyses are not performed. However, we believe that this does not affect the validity of our data, since GA and HbA1c, unlike glycaemic tests, present good pre-analytical stability and less day-to-day variations during stress and illness due to their lifespan. We amended the text for clarity accordingly (**Page 16, paragraph 3, lines 30 – 34**).

**2- GA could have fit to diagnose diabetes in different patient subsets, and COVID-19 is one of them.**

Thank you for this comment. We agree with the reviewer that GA can detect diabetes in different subsets of individuals. As we mentioned previously, data on other subsets are needed for accurate conclusions and adequate clinical use of GA. We believe that our study is the first to assess the performance of GA on admission of hospitalized adults, particularly with COVID-19, and our data offers to the literature more evidence that this test may be useful in different settings to diabetes diagnosis.

**3- It would be nice if the authors could provide their study group’s GA/ HbA1c ratio of their population and its possible clinical implications.**

Thank you for this comment. We understand that evaluating GA/HbA1c ratio clinical implications is beyond the scope of this study. The GA/HbA1c ratio is related to the mean amplitude of glycaemic excursion and is suggested as short-term glycaemic control marker. Nevertheless, as mentioned above, to present indirectly the mean amplitude of glycaemic excursion in our population we provided GA/HbA1c ratio results in **Table 1** in the article file.

Finally, we would like to express our appreciation to you and the reviewers for suggesting how to improve our paper.

Thank you very much,

Yours sincerely,

Joíza Lins Camargo, PhD

Experimental Research Centre

Hospital de Clínicas de Porto Alegre

Rua Ramiro Barcellos, 2350 – 1o andar

Porto Alegre, Brazil 90035-006

e-mail: jcamargo@hcpa.edu.br; Phone +55 51 33598852
